# TROP2 immunoreactivity in pulmonary large cell neuroendocrine carcinoma

**DOI:** 10.1111/his.70226

**Published:** 2026-07-02

**Authors:** Michael Minkley, Kashif Ravasia, Thi Nghiem, Dongxia Gao, Jamie Feng, Andrew Churg, Julia R Naso

**Affiliations:** ^1^ Department of Pathology and Laboratory Medicine University of British Columbia Vancouver British Columbia Canada; ^2^ Undergraduate Medicine University of British Columbia Vancouver British Columbia Canada; ^3^ Department of Clinical Research BC Cancer Vancouver British Columbia Canada; ^4^ Department of Medical Oncology BC Cancer Vancouver British Columbia Canada; ^5^ Department of Pathology and Laboratory Medicine Vancouver General Hospital Vancouver British Columbia Canada

**Keywords:** biomarkers, large cell neuroendocrine carcinoma, pathology, RB1, TROP2

## Abstract

**Introduction:**

TROP2 antibody‐drug conjugates have shown promising anti‐tumour activity, but neither the activity of these agents nor TROP2 expression has been well studied in pulmonary large cell neuroendocrine carcinoma (LCNEC), a rare and aggressive cancer type. We aimed to (i) determine the prevalence of TROP2 immunoreactivity in LCNEC; (ii) assess whether TROP2 immunoreactivity in LCNEC is related to RB1 loss on IHC or to the presence of NSCLC or SCLC components and (iii) determine whether TROP2 immunoreactivity in LCNEC has prognostic significance.

**Methods:**

TROP2 immunohistochemistry was performed on 58 pure LCNECs and 25 areas of LCNEC that were combined with other carcinomas, in tissue microarray format. TROP2 was scored for the percent of tumour cells staining, and with evaluation of cytoplasmic versus membranous staining, approximating a previously published predictive scoring method.

**Results:**

TROP2 staining in ≥1% and ≥50% of LCNEC cells was present in 60% and 26% of pure LCNEC tumours, respectively. Cytoplasmic‐type staining, previously implicated in greater antibody‐drug conjugate response, was present in 48% of pure LCNEC cases. Positive TROP2 staining was less frequent in pure LCNEC than in the LCNEC component of tumours that also contained a NSCLC component (≥1%: *P* = 0.0087; ≥50%: *P* = 0.011), and ≥50% TROP2 staining in pure LCNEC was associated with retained RB1 staining on immunohistochemistry (*P* = 0.032). TROP2 staining was not associated with recurrence‐free survival or disease‐specific survival outcomes.

**Conclusion:**

Frequently positive TROP2 IHC suggests that TROP2 antibody‐drug conjugates may be potential treatments for LCNEC.

## Graphical abstract

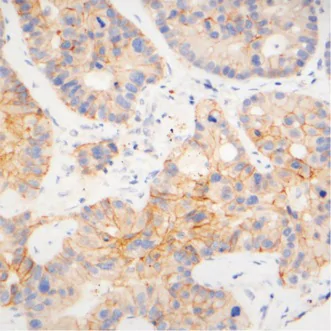

**Abbreviations**
DSS: disease‐specific survival
IHC: immunohistochemistry
LCNEC: large cell neuroendocrine carcinoma
NSCLC: non‐small cell lung carcinoma
RFS: recurrence‐free survival
SCLC: small cell lung carcinoma

## Introduction

Pulmonary large cell neuroendocrine carcinoma (LCNEC) is a rare and aggressive carcinoma in need of novel effective treatment options. Emerging treatment considerations for non‐small cell lung carcinoma (NSCLC) include antibody‐drug conjugates such as TROP2 targeting agents. TROP2 is a cell surface glycoprotein expressed in a variety of carcinomas but with limited expression in most normal tissues.^1^ TROP2 antibody‐drug conjugates have shown promising activity in subsets of NSCLC^1^, ^2^ and small cell lung carcinoma (SCLC)^3^, ^4^, ^5^, ^6^, ^7^, ^8^ but to our knowledge have not yet been trialled in LCNEC.

The selection of patients most likely to benefit from TROP2 antibody‐drug conjugates remains challenging,^2^ fuelling interest in refinement of TROP2 immunohistochemistry (IHC) as a predictive biomarker. A recent digital TROP2 scoring method evaluating membranous versus cytoplasmic expression levels showed predictive value for TROP2 antibody‐drug conjugate datopotamab deruxtecan response.^9^, ^10^, ^11^ Thus, TROP2 IHC, if scored appropriately, may be useful for identifying rare subtypes of lung cancer that may be likely to respond to TROP2 antibody‐drug conjugates, data valuable for clinical trial planning and potentially for off‐label drug use. The predictive scoring system (QCS‐NMR) was based on membranous TROP2 expression compared with total cytoplasmic plus membranous TROP2, with the group having less than 56% of the staining in the membrane compartment (specifically, ≥75% of tumour cells with normalized membrane ratio ≤0.56) associated with greater drug response.^9^, ^10^ Exact recapitulation of the previously reported predictive scoring system is not possible as it incorporated the authors' internal deep learning algorithms; however, their methodology may be approximated by manually considering cytoplasmic versus membranous localization of staining. To our knowledge, an approximation of this scoring system has not previously been applied to lung carcinoma subtypes beyond those in the datopotamab deruxtecan clinical trials.^9^, ^10^, ^11^

In addition to having predictive value for treatment response, TROP2 immunoreactivity is a poor prognostic factor in multiple other cancer types.^12^, ^13^ However, neither the prognostic significance of TROP2 in LCNEC, nor its association with LCNEC molecular subgroups^14^ or combined histologic components, has been assessed. LCNEC may comprise the entirety of a tumour (‘pure LCNEC’), may be combined with another non‐small cell lung carcinoma (NSCLC) component (‘combined LCNEC’) or may be combined with small cell carcinoma (SCLC, ‘combined SCLC’).

In this study, we aimed to (i) determine the prevalence of TROP2 immunoreactivity (including cytoplasmic‐predominant staining assessment) in LCNEC; (ii) assess whether TROP2 immunoreactivity in LCNEC is related to retained RB1 IHC staining or to the presence of NSCLC or SCLC components and (iii) determine whether TROP2 immunoreactivity in LCNEC has prognostic significance. TROP2 had at least focal staining in the majority of pure LCNEC cases, promising for consideration of TROP2 antibody‐drug conjugate trials in LCNEC.

## Materials and methods

### Study Cohort

Research Ethics Board approval for this study was received from the University of British Columbia (H24‐01406). Cases with at least a component of LCNEC were identified from excision specimens in clinical archives and diagnoses were confirmed according to WHO 5th edition criteria.^15^ Staging used the AJCC 9th edition.^16^ Four combined LCNEC cases were excluded as the area of LCNEC was too small for representation in the tissue microarray. At least focal (≥1%) staining with synaptophysin, chromogranin or INSM1 was present in all included cases (three cases were only CD56 positive and were excluded). Diffuse neuroendocrine marker staining was defined as staining with INSM1, synaptophysin or chromogranin in ≥20% of carcinoma cells, a threshold used previously.^14^ Three patients who had prior lung adenocarcinoma treated with *EGFR*‐targeted agents were excluded to exclude treatment‐related neuroendocrine transformations. Survival outcome data were obtained through retrospective chart review. Pure LCNEC cases in this study are further described in a prior publication.^17^

### Immunohistochemistry

IHC was performed on tissue microarrays containing two 1 mm cores of the LCNEC component of each case, such that our TROP2 assessment was exclusively in the histologically LCNEC areas of the combined tumours (i.e. NSCLC areas were not assessed in the tissue microarrays). TROP2 IHC (Clone ENZ‐ABS380‐0100, 1:1500 dilution, 30 min incubation, Cedarlane, Burlington, CA) was performed on a BOND Rx autostainer platform (Leica), with heat‐induced epitope retrieval using Epitope Retrieval 2 (Leica) for 20 min. Staining was visualized using the BOND Polymer Refine Detection kit (Leica).

TROP2 cytoplasmic or membranous staining of any intensity in ≥1% tumour cells was considered positive. Positive staining cases were subdivided based on whether <25% of positive carcinoma cells had more membranous than cytoplasmic staining, simulating a recent approach applied to NSCLC.^9^, ^10^ If a cell membrane could be appreciated on the immunostain, that cell was considered to have more membranous than cytoplasmic staining, on the basis of stronger intensity at the membrane. TROP2 staining was independently scored by two pathologists blinded to each other's scores, and discrepant cases were reviewed together to achieve consensus. Other IHC was performed and scored as described in Table S1.

### Statistical Analysis

Relationships between categorical variables were assessed using chi‐squared tests if the expected frequency for all categories was ≥5; otherwise, Fisher's Exact test was used. Cox proportional hazards models were used for survival analysis. Associations with block age were tested using Wilcoxon rank‐sum tests. Interobserver agreement was calculated using Cohen's kappa (*κ*) with 95% confidence intervals (CI). Statistical analysis was performed in R.^18^ *P* < 0.05 were considered statistically significant.

## Results

### Study Cohort

The study cohort consisted of 58 pure LCNECs, 17 combined LCNECs with non‐small cell carcinoma components (13 with adenocarcinoma, 2 with squamous cell carcinoma, 1 with adenocarcinoma and squamous cell carcinoma, and 1 with large cell carcinoma), 6 combined SCLCs with LCNEC components and 2 tumours with LCNEC, SCLC and adenocarcinoma components. Median year of diagnosis was 2016 (range 1997–2024, IQR 11.5 years) and median patient age was 69 years (range 43–85 years, IQR 11 years). All specimens were from resection/excision procedures. The pure LCNEC included early‐stage resections plus specimens from nine patients with advanced stage disease at the time of specimen excision (four excisions of brain metastases, two excisions of groin metastases, one excision of salivary gland metastasis and two patients with their primary lung tumour excised following advanced stage disease identification). Clinical and pathology features of the cohorts are summarized in Table 1 (pure LCNEC) and Supplemental Table S2 (combined LCNEC).

**Table 1:** Pure LCNEC cohort clinical and pathology features

Feature	Pure LCNEC (total *n* = 58) [*n* (%)]
Male	27 (47)
Smoking history	
Never	2 (3)
Former/current	48 (83)
No data	8 (14)
Stage at diagnosis	
IA	17 (29)
IB	8 (14)
IIA	8 (14)
IIB	10 (17)
IIIA	6 (10)
IV	9 (16)
Complete resection	47 (81)
Adjuvant chemotherapy	16 (28)
Chemotherapy for advanced stage	15 (26)
TTF1 positive	37 (63)
Diffuse neuroendocrine marker staining^a^	50 (86)
Focal neuroendocrine marker staining^b^	8 (14)

^a^ Staining with INSM1, synaptophysin or chromogranin in ≥20% of carcinoma cells.

^b^ Staining with INSM1, synaptophysin or chromogranin in <20% of carcinoma cells.

### Prevalence of TROP2 Immunostaining

Among the 58 pure LCNEC tumours, cytoplasmic or membranous TROP2 staining of any intensity in ≥1% of tumour cells was detected in 35 of the 58 cases (60%) and in ≥50% of tumour cells in 15 of 58 cases (26%). Three cases had very focal staining in <1% of tumour cells, and the remaining 20 cases had no staining detected. Whole slide sections of 8 pure LCNEC tumours (five tumours with <1%, 2 tumours with 1%–49% and 1 tumour with ≥50% TROP2 staining on the tissue microarray) showed TROP2 immunostaining in the same category as on the tissue microarray (Figure S1).

TROP2 staining was less frequent in pure LCNEC than in the LCNEC component of mixed tumours with a NSCLC component (≥1% threshold: 60% vs. 94%, *P* = 0.0087; ≥50% threshold: 26% vs. 59%, *P* = 0.011, Table S2). As combined components may not have been detectable in excised tissue from advanced stage cases, we also assessed for association of TROP2 positivity with combined NSCLC histology among just the early‐stage cases and again found this association (≥1% threshold: *P* = 0.027; ≥50% threshold: *P* = 0.025). Whole slide sections of five LCNEC combined with NSCLC showed ≥50% staining in the NSCLC component and less diffuse staining in the LCNEC component on the same section (one <1%, four 1%–49%, Figure S2).

There was excellent agreement between observers at both thresholds (1% threshold: *κ* = 0.81, 95% CI 0.67–0.94; 50% threshold: *κ* = 0.89, 95% CI 0.79–0.99; full 83 case cohort for this analysis). An association of <1% and <50% staining with older block age was identified (*P* = 0.0093 and 0.0017; Figure S3), such that the overall cohort analysis may slightly underestimate the prevalence of TROP2 staining in LCNEC. Among pure LCNEC cases with block age less than or equal to the median of 9 years, 23 of 31 (74%) showed positive (≥1%) TROP2 staining and 12 of 31 (39%) showed diffuse (≥50%) TROP2 staining. There was no significant difference in block age between pure LCNEC and LCNEC combined with NSCLC (*P* = 0.62; blocks ≤9 years old for 31/58 pure cases vs. 11/17 cases combined with NSCLC).

### Association of TROP2 Staining with Pathologic and Clinical Features of Pure LCNEC

We then focused analyses on only the pure LCNEC group. TROP2 positivity was more frequent in tumours with focal rather than diffuse neuroendocrine marker staining (*P* = 0.017 for ≥1% TROP2 staining; *P* = 0.022 for ≥50% TROP2 staining, Table 2), with no significant association of diffuse neuroendocrine marker staining with block age (P = 0.51). Molecular subgroups of LCNEC^14^ were approximated using RB1 IHC, scored as lost or retained. TROP2 ≥ 50% staining was associated with retained RB1 staining on IHC (P = 0.032), whereas TROP2 ≥ 1% was not (*P* = 0.30, Table 2).

**Table 2:** TROP2 immunoreactivity in pure LCNEC, in relation to RB1 IHC and neuroendocrine marker staining

Feature	Number in pure LCNEC cohort (*n* [%])	TROP2 ≥ 1% staining (*n* [%])	TROP2 ≥ 50% staining (*n* [%])	TROP2 cytoplasmic‐type staining (*n* [%])
Total among pure LCNEC cases	58	35	15	28
RB1 IHC staining				
Retained	25 (43)	17 (49)	10 (67)	13 (46)
Lost	33 (57)	18 (51)	5 (33)	15 (54)
Neuroendocrine marker staining^a^				
Diffuse	50 (86)	27 (77)	10 (67)	23 (82)
Focal	8 (14)	8 (23)	5 (33)	5 (18)

SCLC—small cell carcinoma. NSCLC—non‐small cell carcinoma. IHC—immunohistochemistry. Percent indicates the percent of the total at the top of each column.

^a^ Staining with INSM1, synaptophysin or chromogranin in <20% of carcinoma cells.

There was no significant difference in positive TROP2 staining between the 49 early and 9 advanced stage cases (≥1% threshold: 65% vs. 33% positive, *P* = 0.13; ≥50% threshold: 29% vs 11% positive, *P* = 0.42). To further compare TROP2 staining in early and advanced stage disease, we performed whole slide section staining on three matched primary and metastasis pairs (all pure LCNEC with the primary tumour already in the cohort and with later excision of brain metastases). These showed TROP2 staining in the same category as the primary (≥50% for one, and 1%–49% for two; Figure S1).

Cases with ≥1% staining were then scored in a manner approximating a recent scoring system predictive of response to a TROP2‐directed antibody‐drug conjugate in NSCLC.^9^, ^10^ Of the 58 pure LCNEC cases, 7 (12%) had ≥25% of tumour cells with more membranous than cytoplasmic staining (‘membranous‐type’ staining), and 28 (48%) had <25% of cells with more membranous than cytoplasmic staining (‘cytoplasmic‐type’ staining, Figure 1). There was excellent interobserver agreement for cytoplasmic versus membranous‐type staining distinction (*κ* = 0.84, 95% CI 0.62–1.00). Cytoplasmic versus membranous‐type staining was not associated with retained RB1 staining on IHC (*P* = 0.69), focal neuroendocrine marker staining (*P* = 0.30) or block age (*P* = 0.24).

**Figure 1:**
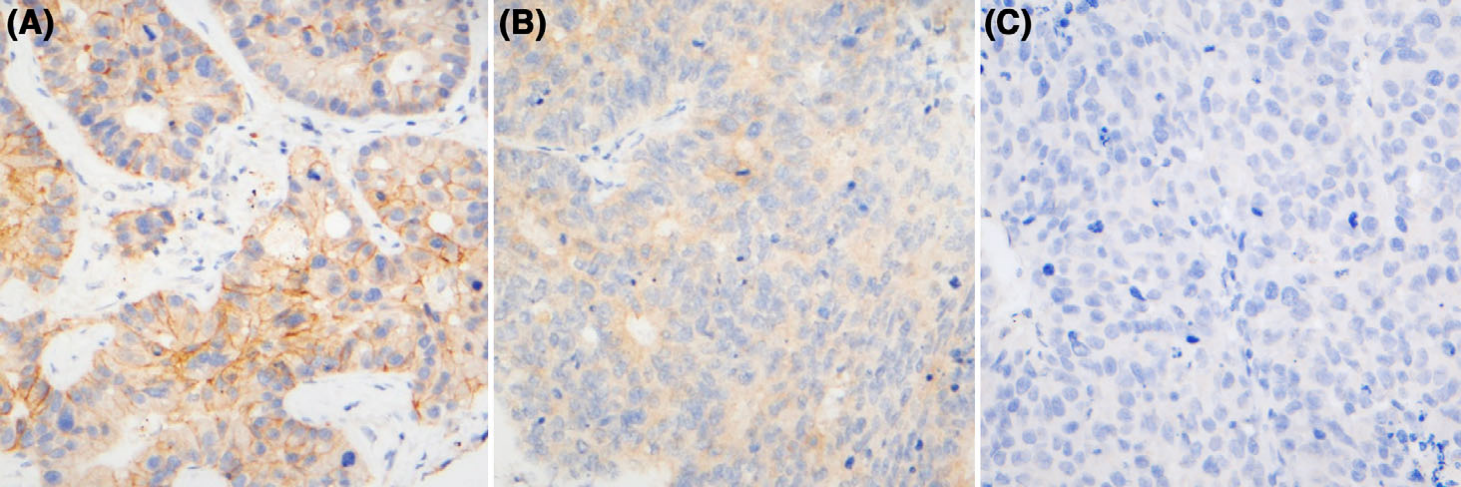
TROP2 IHC with (**A**) membranous‐type, (**B**) cytoplasmic‐type and (**C**) negative (<1%) staining in LCNEC.

Forty‐seven patients with resected early‐stage disease had sufficient data for recurrence‐free survival (RFS) analysis from the time of resection, of whom 26 (55%) had recurrence. RFS from the date of resection was not associated with TROP2 ≥ 1% (HR 0.96, 95% CI 0.41–2.26, *P* = 0.93), TROP2 ≥ 50% (HR 1.73, 95% CI 0.75–4.00, *P* = 0.20) or TROP2 cytoplasmic‐type staining (HR 0.81, 95% CI 0.36–1.80, *P* = 0.60, Figure S4). Thirty‐one patients who developed advanced stage disease had sufficient data for disease‐specific survival (DSS) analysis, of whom 28 died of disease. DSS from the date of advanced stage disease identification was not associated with TROP2 ≥ 1% (HR 0.92, 95% CI 0.42–2.00, *P* = 0.83), TROP2 ≥ 50% (HR 1.31, 95% CI 0.53–3.23, *P* = 0.55) or TROP2 cytoplasmic‐type staining (HR 1.06, 95% CI 0.49–2.30, *P* = 0.89). Adjusting for block age in multivariable analysis did not produce significant associations with RFS or DSS (all *P* ≥ 0.21).

## Discussion

In this study, we provide the first assessment of TROP2 expression in LCNEC in relation to histologic, IHC and clinical outcome features. TROP2 staining was present in a considerable proportion of pure LCNEC tumours (60% with ≥1% tumour cell staining and 26% with ≥50% staining, with frequent cytoplasmic‐type staining among positive cases), providing a rationale for investigation of TROP2 antibody‐drug conjugate response in LCNEC.

TROP2 overexpression has been associated with prognosis in multiple malignancies, with variable survival associations depending on the histologic type.^13^ In a prior study, high‐level TROP2 membranous IHC staining was associated with worse prognosis in lung adenocarcinoma, but better prognosis in a high‐grade lung neuroendocrine carcinoma group, in which 41 out of 115 patients had LCNEC.^12^ Analysis specifically of the LCNEC subgroup was not provided in that report. The present study did not detect association of TROP2 with survival outcomes of patients with pure LCNEC.

To our knowledge, the aforementioned study analysing high grade neuroendocrine carcinomas is the only prior study of TROP2 IHC in pulmonary LCNEC, and scored only membranous staining. Low level staining was not distinguished from entirely negative staining in this prior study, which used only tissue microarrays and included blocks from a 24 year span (similar to the time range in our study). High‐level staining was reported in 14 out of 41 cases (34%; similar to our finding of 26% of pure LCNEC cases having ≥50% staining), and high‐level TROP2 staining in LCNEC was more prevalent than in SCLC but less prevalent than in NSCLC.^12^ Similar to the greater TROP2 staining we observed in LCNEC with focal rather than diffuse neuroendocrine marker staining, TROP2 expression was enriched in POU2F3 positive SCLC tumours, which typically have minimal neuroendocrine marker expression.^5^, ^6^

Scoring systems for TROP2 IHC are highly variable,^13^ and to our knowledge, this study is the first to attempt to simulate a digital TROP2 scoring system that has been shown to correlate with treatment response^9^, ^10^ and produce reproducible scores across sites,^11^ through distinction of cytoplasmic versus membranous staining. Cytoplasmic staining may reflect internalization of TROP2 proteins transiently at the membrane, potentially enhancing cytotoxic activity of antibody‐drug conjugates internalized with TROP2, though this hypothesis remains to be tested.

The limited methodological detail available for the digital method (published only as abstracts^9^, ^10^, ^11^) makes externally testing the generalizability of their approach challenging. Training artificial intelligence recognition of membrane vs cytoplasmic staining is not trivial, as conventional cell segmentation algorithms approximate cell boundaries using distances between nuclei, which often poorly overlap with the actual cell membrane. However, human observers can readily distinguish membranous and cytoplasmic staining, such that a manual approach was used in this study. In the absence of treatment response data, whether our scoring retains predictive value is uncertain. The frequent cytoplasmic‐type staining we identify is nonetheless encouraging for testing TROP2 antibody‐drug conjugates in LCNEC, which would generate outcome data to which IHC scores could be compared.

Additional limitations of this study include its use of retrospective data and the use of two tissue cores per tumour for IHC, which may not fully capture heterogeneity within the tumours and only represented the LCNEC component of combined tumours. Our cohort predominantly represented resectable early‐stage disease, due to challenges in confident diagnosis of LCNEC on small biopsy specimens. The possible loss of antigenicity over time in tissue blocks is a challenge for TROP2 studies on rare archival material, and true TROP2 prevalence in LCNEC may be even greater than estimated in our study. Our detection of frequent TROP2 staining despite the archival nature of the tissue establishes that at minimum TROP2 has at least moderately frequent immunostaining in LCNEC.

This study provides supporting rationale for investigation of TROP2 antibody‐drug conjugates in patients with LCNEC. Further exploration of novel biomarkers predictive of targeted therapy response in LCNEC may expand treatment options and improve outcomes for patients with this aggressive and rare carcinoma.

## Author contributions

MM was involved in data curation, formal analysis and data visualization. KR and DG assisted with data curation. TN supported formal analysis and methodology selection. JF was involved in conceptualization, resources acquisition and methodology. AMC assisted with project administration, resource acquisition, investigation and funding acquisition. JRN was involved in conceptualization, data curation, formal analysis, funding acquisition, project administration, supervision, visualization and original draft preparation. All authors assisted with manuscript review and editing, and have read and approved the final manuscript.

## Conflicts of interest

JRN has received consulting fees from Astra‐Zeneca and Bayer. For the remaining authors, none were declared.

## Funding information

This project received funding from the University of British Columbia Diagnostic and Molecular Pathology Residency Program.

## Supporting information


**Figure S1.** TROP2 immunohistochemistry on pure LCNEC whole slide sections. (A–C) Patients with <1% TROP2 staining on the TMA also had <1% TROP2 staining on whole slide sections. (D–L) Patients with at least 1% TROP2 staining in their primary tumours had a similar extent of staining in their subsequent metastatic disease. Row labels indicate whether the primary tumour or metastatic tumour (brain metastasis) is shown and the objective used for imaging.
**Figure S2:** TROP2 and synaptophysin immunohistochemistry on whole slide sections of combined LCNEC with an adenocarcinoma component. Shown are fields with LCNEC in the top/left (synaptophysin positive) and adenocarcinoma on the bottom/right (synaptophysin negative). In these examples, the LCNEC component has more focal TROP2 staining than the adjacent adenocarcinoma component.
**Figure S3:** Tissue block age was associated with negative (<1%) TROP2 on immunohistochemistry (IHC), when including blocks older than 15 years. Cytoplasmic versus membranous TROP2 staining patterns were not significantly associated with block age (NA: not applicable for pattern scoring, as <1% TROP2 staining). Plots include the full 83 patient cohort (pure LCNEC as well as LCNEC combined with other histologic types).
**Figure S4:** Kaplan–Meier curves of (A) recurrence‐free survival and (B) disease‐specific survival of stratified by TROP2 IHC results. Only cases with pure LCNEC are included. P‐values are from univariable Cox Proportional Hazards Models.

**Table S1.** Immunohistochemistry protocols, using a Dako OMNIS platform (Agilent Technologies, Santa Clara, USA) and Envision Flex detection system (Agilent Technologies).
**Table S2:** Clinical and pathology features of cases with mixed histologic features.

## Data Availability

The data will be made available upon reasonable request.
