# Supplementary material for: TROP2 immunoreactivity in pulmonary large cell neuroendocrine carcinoma
Source: Histopathology. 2026 Jul 2;89(5):779–85. doi: 10.1111/his.70226 (PMC13643618; doi:10.1111/his.70226)
Supplement: Supplementary file 1 [file HIS-89-779-s001.docx]

**Supplemental Table S1**: Immunohistochemistry protocols, using a Dako OMNIS platform (Agilent Technologies, Santa Clara, USA) and Envision Flex detection system (Agilent Technologies).

| **Antibody** | **Clone** | **Vendor** | **Retrieval Buffer pH** | **Dilution** | **HIER Time** | **Antibody (min)** | **Linker (min)** | **Polymer (min)** | **Scoring** |
| --- | --- | --- | --- | --- | --- | --- | --- | --- | --- |
| TTF1 | 8G7G3/1 | Dako | High | 1:200 | 30 | 30 | 10 | 30 | Any nuclear staining is ‘positive’ |
| Synaptophysin | DAK-SYNAP | Dako | High | RTU | 30 | 20 | None | 20 | Percent of carcinoma cells with cytoplasmic staining |
| Chromogranin | LK2H10 | Cell Marque | High | 1:200 | 30 | 30 | None | 30 | Percent of carcinoma cells with cytoplasmic staining |
| INSM1 | MRQ-70 | Cell Marque | High | 1:25 | 30 | 30 | None | 30 | Percent of carcinoma cells with nuclear staining |
| RB1 | 13A10 | Leica | High | 1:50 | 30 | 30 | 10 | 25 | Any nuclear staining is ‘retained’ |

**Supplemental Table S2:** Clinical and pathology features of cases with mixed histologic features

| **Feature** | **LCNEC Combined with NSCLC**  **(Total n=17)**  **[n (%)]** | **SCLC Combined with LCNEC**  **(Total n=6)**  **[n (%)]** | **SCLC Combined with LCNEC and NSCLC**  **(Total n=2)**  **[n (%)]** |
| --- | --- | --- | --- |
| Male | 8 (47) | 3 (50) | 1 (50) |
| Smoking history |  |  |  |
| Never | 0 (0) | 0 (0) | 0 (0) |
| Former/current | 17 (100) | 6 (100) | 2 (100) |
| No data | 0 (0) | 0 (0) | 0 (100) |
| Stage at diagnosis |  |  |  |
| IA | 6 (35) | 2 (33) | 2 (100) |
| IB | 4 (24) | 3 (50) | 0 (0) |
| IIA | 1 (6) | 0 (0) | 0 (0) |
| IIB | 4 (24) | 1 (17) | 0 (0) |
| IIIA | 2 (12) | 0 (0) | 0 (0) |
| IV | 0 (0) | 0 (0) | 0 (0) |
| Complete resection | 17 (100) | 3 (50) | 2 (100) |
| Adjuvant chemotherapy | 5 (29) | 5 (83) | 1 (50) |
| Chemotherapy for advanced stage | 5 (29) | 1 (17) | 0 (0) |
| TTF1 positive | 12 (71) | 5 (83) | 1 (50) |
| Diffuse neuroendocrine marker staining^1^ | 14 (82) | 4 (67) | 2 (100) |
| Focal neuroendocrine marker staining^2^ | 3 (18) | 2 (33) | 0 (0) |
| **TROP2 ≥ 1% staining** | 16 (94) | 4 (67) | 1 (50) |
| **TROP2 ≥ 50% staining** | 10 (59) | 2 (33) | 1 (50) |
| **TROP2 cytoplasmic type staining** | 14 (82) | 3 (50) | 1 (50) |

^1^Staining with INSM1, synaptophysin or chromogranin in ≥ 20% of carcinoma cells.

^2^Staining with INSM1, synaptophysin or chromogranin in < 20% of carcinoma cells.
