# Supplementary material for: TROP2 immunoreactivity in pulmonary large cell neuroendocrine carcinoma
Source: Histopathology. 2026 Jul 2;89(5):779–85. doi: 10.1111/his.70226 (PMC13643618; doi:10.1111/his.70226)
Supplement: Supplementary file 2 [file HIS-89-779-s002.docx]

**Supplemental Figure S1:** TROP2 immunohistochemistry on pure LCNEC whole slide sections. (A-C) Patients with <1% TROP2 staining on the TMA also had <1% TROP2 staining on whole slide sections. (D-L) Patients with at least 1% TROP2 staining in their primary tumors had a similar extent of staining in their subsequent metastatic disease. Row labels indicate whether the primary tumor or metastatic tumor (brain metastasis) is shown, and the objective used for imaging.

**Supplemental Figure S2:** TROP2 and synaptophysin immunohistochemistry on whole slide sections of combined LCNEC with an adenocarcinoma component. Shown are fields with LCNEC in the top/left (synaptophysin positive) and adenocarcinoma on the bottom/right (synaptophysin negative). In these examples, the LCNEC component has more focal TROP2 staining than the adjacent adenocarcinoma component. (20x objective).

**Supplemental Figure S3:** Tissue block age was associated with negative (<1%) TROP2 on immunohistochemistry (IHC), when including blocks older than 15 years. Cytoplasmic versus membranous TROP2 staining patterns were not significantly associated with block age (NA: not applicable for pattern scoring, as <1% TROP2 staining). Plots include the full 83 patient cohort (pure LCNEC as well as LCNEC combined with other histologic types).

**Supplemental Figure S4:** Kaplan-Meier curves of (A) recurrence-free survival and (B) disease specific survival of stratified by TROP2 IHC results. Only cases with pure LCNEC are included. P-values are from univariable Cox Proportional Hazards Models.
